# High Accuracy WiFi-Based Human Activity Classification System with Time-Frequency Diagram CNN Method for Different Places

**DOI:** 10.3390/s21113797

**Published:** 2021-05-30

**Authors:** Lokesh Sharma, Chung-Hao Chao, Shih-Lin Wu, Mei-Chen Li

**Affiliations:** 1 Department of Computer Science and Information Engineering, Chang Gung University, Taoyuan 33302, Taiwan; d000015472@cgu.edu.tw (L.S.); m0629016@cgu.edu.tw (C.-H.C.); mcli@cgu.edu.tw (M.-C.L.); 2 Department of Cardiology, Chang Gung Memorial Hospital, Taoyuan 33305, Taiwan; 3 Department of Electrical Engineering, Ming Chi University of Technology, New Taipei City 24301, Taiwan

**Keywords:** fall detection, channel state information, wireless, device-free, different place

## Abstract

Older people are very likely to fall, which is a significant threat to the health. However, falls are preventable and are not necessarily an inevitable part of aging. Many different fall detection systems have been developed to help people avoid falling. However, traditional systems based on wearable devices or image recognition-based have many disadvantages, such as user-unfriendly, privacy issues. Recently, WiFi-based fall detection systems try to solve the above problems. However, there is a common problem of reduced accuracy. Since the system is trained at the original signal collecting/training place, however, the application is at a different place. The proposed solution only extracts the features of the changed signal, which is caused by a specific human action. To implement this, we used Channel State Information (CSI) to train Convolutional Neural Networks (CNNs) and further classify the action. We have designed a prototype to test the performance of our proposed method. Our simulation results show an average accuracy of same place and different place is 93.2% and 90.3%, respectively.

## 1. Introduction

According to the US Centers for Disease Control and Prevention, one out of every four elder people over the age of 65 years has fallen once in a year, and less than half of them have not informed their doctors. Furthermore, the risk of falling again will be double if you have fallen once [1]. Falling is a dangerous situation for the elderly, and it can result in fractures, internal bleeding, and death in severe cases. Furthermore, the expenses of fall medical care are a major annual expenditure for a country. For example, in 2015, the United States used more than 50 billion USD in medical expenses for falls [1]. Because of the above reasons, a precise and convenient fall detection system is essential.

In the past, many fall detection systems have been proposed based on different methods, such as systems based on wearable sensing devices [2,3], image recognition [4,5,6,7], smartphones [8,9], etc. However, these systems also have some problems. Systems based on wearable sensing devices are not user-friendly. Older people usually forget to wear them. Systems based on image recognition have a privacy issue since they need continuous monitoring by the camera, and in a dark environment, action cannot be recognized correctly by the ordinary camera. However, the 3D active vision camera can identify them in the dark. Systems based on a smartphone are inconvenient since all the time users need to carry them. Due to the following problems, only a small number of fall detection systems have real-life application.

References [10,11] survey elderly fall detection. Combining sensor networks and IoT shows that fusing the wireless signals and signals of different sensors could result in higher accuracy and lower false alarms. The study [11] identified challenges of usability, user acceptance, power consumption, sensing limitations, privacy. Due to the popularity of wireless network technology in recent years, we can easily receive more detailed Channel State Information (CSI) at the physical layer from a specific network interface card (NIC) [12]. One of the most obvious advantages of using CSI to detect human motion is that it does not have all the shortcomings of traditional fall detection systems. Therefore, in recent years, a lot of researches have been designed to detect falls [13,14,15,16,17,18], actions [19,20], gestures [21], identity [22,23], user input on the keyboard [24], and so on. Some of the above systems use deep learning techniques to recognize falls [16] and actions [19,20]. However, these deep learning methods directly take the raw CSI or amplitude/phase information of CSI as input. These methods can improve the accuracy while testing at the same place. However, the same method at different places shows poor accuracy [17].

CSI is very sensitive to the changes; measurements at different times may have different results if the environment of the place is not static. The static environment means that nothing is moving except the measurement object. The difference of measured CSIs at a different place is very significant. Due to this, many studies have shown poor accuracy. As a result, these kinds of systems are not commercialized [15,19]. The motivation of our research is to solve this problem. Our main idea is that the same action performed at different places, has a similar CSI time-frequency diagram in the static environment [17] which is very suitable for elderly people living alone. Therefore, compared with [19,20] which take the CSI information directly as input for deep learning, our proposed solution extracts the features by the Convolutional Neural Networks (CNNs) model first and considers the time-frequency graph, which increases the accuracy. This diminishes the effects of testing the system in a new environment rather than a training environment. Therefore, our solution shows more robustness while achieving better accuracy.

The remaining paper is organized as follows. In Section 2, we discuss about the related works. Section 3, discusses about the preliminaries that we have used in the paper such as received signal strength indicator (RSSI) and CSI. In Section 4, the architecture and proposed method is described. Section 5 discusses the system performance and Section 6 conclude our paper and propose future directions.

## 2. Related Work

In related work, indoor fall detection systems can be classified according to the sensors used and WiFi signal processing mechanisms. The sensor-based fall detection system is entirely dependent on the hardware, such as a wearable computer or smartphone, while the WiFi signal processing can be categorized into two types: RSSI-based and CSI-based. In this part, we will go through the studies that are focused on them.

### 2.1. Sensors Based Fall Detection

References [2,3] have used sensor to detect the fall. In study [2], a barometric pressure sensor is used to aid in the discrimination of true dropping cases. The user’s acceleration and air pressure data are constantly tracked using a wearable tracker connected to the user’s waist. The suggested device has a 96.9% percent accuracy. The paper [3] proposes a crossbow wireless sensor node device for detecting human falls. The tri-axis accelerometer and the Passive Infrared sensor detect falls. The sensors sense changes in voltage in real-time.

Study [8,9] proposes a pervasive fall detection system based on smartphone. Based on an accelerator sensor, which aids in the calculation of PerFallID. However, the above methods demanded the use of special hardware that must be worn or carried at all times.

### 2.2. Image Based Fall Detection

In study [7], authors have focused on vision-based methods based on a single RGB camera, 3D methods for multiple RGB cameras, and 3D methods using depth cameras. In references [4,5] authors suggest an optimized time motion images and eigenspace to derive eigen-motion together in this analysis. In addition, MLP Neural Network is used to accurately classify motion and determine a falling event. The average recognition score is 89.99%, according to the experimental results. The authors of the study [6] have used a motion detector asynchronous temporal contrast vision sensor that gathers temporal resolution for sub-milliseconds. To protect the users’ privacy, a non-intrusive mechanism of high precision is proposed.

### 2.3. Multiple Sensors Based Fall Detection

In the study [25,26], the authors have used a multimodal approach, in which the information has been collected from multiple sources such as cameras, microphones, wearable sensors, ambient sensors, smart devices. The combined information helps to improve the classification. For motion classification and identification, CNN has implemented. References [27,28,29], determine the action in the dark these studies need some prerequisites such as initial floor plane information, Kinect sensor, 3D camera. The study [27] requires initial floor plane information and a 3D camera for identification. The study [28] required a Kinect sensor for a 3D depth image. From our understanding, the 3D approach needs more scale of cameras [29] or specific installation of hardware. However, various strategies and expertise are needed to develop and set up a multimodal all detection system. The complex structure, procedure, and cost of this approach make setup challenging.

Many research [30,31,32] has employed 3D vision to identify or prevent falls by looking at head trajectories, body form changes, or body posture. Study [30] has implemented the most basic vision-based method that uses a single camera and the k-Nearest Neighbours (kNN) algorithm to assess silhouette change over time with a camera module, which is the cheapest system in this category and is straightforward to set up; however, because of the restricted area coverage, the performance of such an approach has to be improved. The authors of the study [31] use optical flow to include motion information, and they use a 3D CNN to extract both spatial and temporal data, making it a comprehensive and obvious choice for motion recognition. To include visual attention and find the region of interest, the authors use Long short-term memory (LSTM) with 2D CNN. The authors of the study [32] have used stereo camera information, to estimate the human position and ground plane in 3D. Since detecting a fall may be obtained from knowledge-based reasoning, the human stance is estimated using a CNN to identify a fall based on basic hand-crafted attributes. However, some specific devices, such as RGB camera(s), stereo camera(s), depth camera(s) (Kinect), the thermal sensor(s), or even several camera combinations, were required in the above-mentioned studies.

### 2.4. Wifi Based Fall Detection

One of the first significant research results on WiFi-based sensings for user positioning and tracking was the [33] report. WiFi RSSI has been used for indoor localization in this study. The localization has since depended upon data from WiFi RSSI. The RSSI has been commonly used to localization, human activity recognition [34] and for gesture recognition [35]. RSSI is easy to use to identify human activity identification, but it suffers from fading, severe distortions and inconsistency in a dynamical setting. However, RSSI is not benefiting from Orthogonal Frequency Division Multiplexing (OFDM) subcarrier networks [36].

The CSI based studies [13,17,19,20] has proposed different models while considering the complex features of CSI and consider machine learning approaches for classification. In study [17] authors proposes a CSI based human Activity Recognition and Monitoring system (CARM). The authors try to exploit a CSI-speed model and CSI-activity model to quantifies the correlation between the movement speeds of different human body parts and a specific human activity. In light of the actuality that diverse human developments will cause extraordinary speed changes, CARM evaluates the relationship between’s speed and development, and perceives a given action with Hidden Markov Model.The average accuracy of CARM is greater than 96%. The paper [19], integrates the amplitude and phase of CSI to propose a hybrid complex feature for deep-learning-based approach that monitors the wireless signals. Machine learning and deep learning methods have implemented which shows that by using 6% of training samples, the hybrid feature still achieves 93% accuracy. In literature [20], authors propose a learning method that analyzes the CSI of multiple Access Point (AP)s in a small area to detect and recognize human activities by using deep learning models.

Literature [37,38] have discussed the usage of radar technology for detecting falls. Study [37], proposes CapsFall, based on Ultra-Wideband (UWB) radar which relies on multi-level feature learning from radar time-frequency representations. A capsule network for automating feature learning and enhance model discriminability. The proposed solution outperforms the other methods. Paper [38] propose a learning model that combines convolutional layers and convolutional long short term memory (ConvLSTM) to extract robust spatiotemporal features for fall detection by using monostatic radar. In a word, UWB requires some specific hardware devices to detect falls and is not common in general households compared with WiFi devices.

However, the robustness of the model is not evaluated in a static and dynamic environment. In study [13] authors proposes WiFall, a physical layer CSI as the indicator of activities. Detect fall of the human without hardware modification, extra environmental setup, or any wearable devices. Authors have used motion detection by anomaly detection algorithm, and further, classify the human activities by Random Forest algorithm. The results achieve an average of 94% fall detection precisions.

As far as we know, Wifall [13] is the first to use WiFi signals to detect falls. At the time, deep learning was not popular nowadays. Therefore, many studies were inspired by “Wifall” and did not use deep learning to classify actions until recently. More and more studies use deep learning to classify human activities, but even if these studies use deep learning to achieve action classification, they still not be able to overcome the problem of training models adapting to the new environment.

## 3. Preliminaries

**Figure 1:**
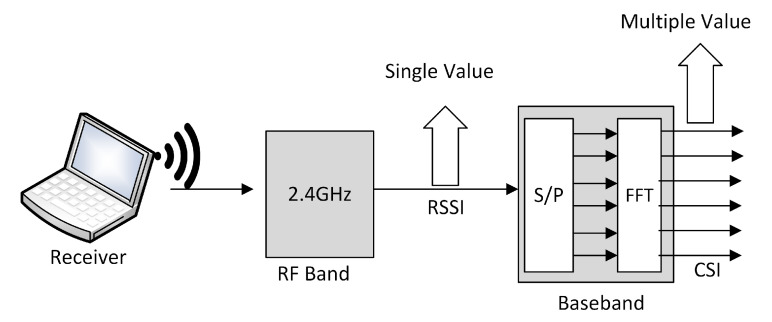
Frequency diversity in RSSI and CSI.

In this section, first, we introduce the RSSI and how it is used for location estimation. However, it is not reliable as it shows adverse effects in different temperatures, humidity. Later, we discuss the CSI and its properties in detail. In Figure 1, we show the RSSI is a single value, whereas the CSI has multiple values due to the Baseband processing. Which shows that the CSI values are frequency diversified.

### 3.1. Received Signal Strength Indicator (RSSI)

**Figure 2:**
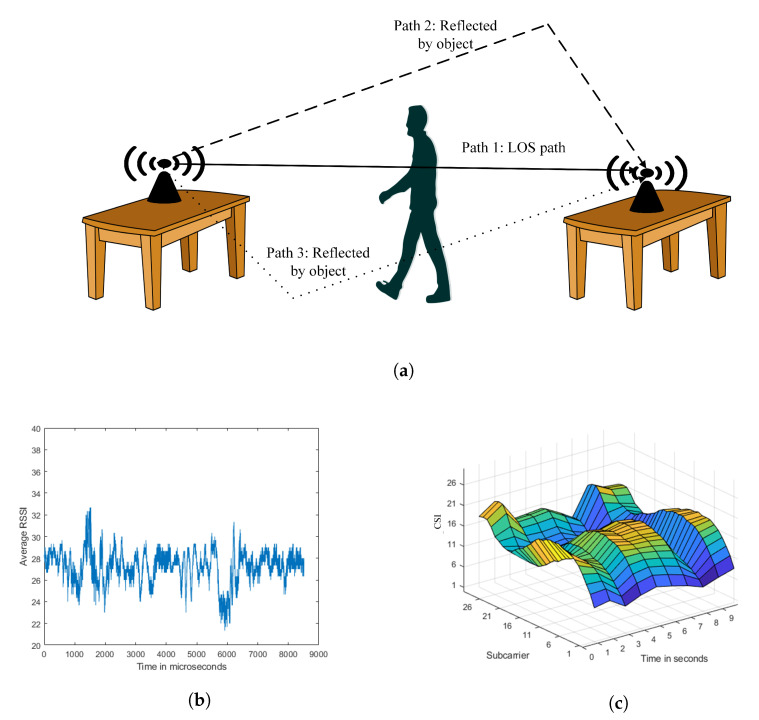
(**a**) An environment with different paths modulated in the WiFi signals; (**b**) average RSSI; (**c**) CSI for 30 sub-carriers.

WiFi is from the family of IEEE 802.11(a/b/g/n/ac) standard protocols, all are the wireless local network technologies. For wireless data transmission, a WiFi AP can connect multiple devices such as laptops, smartphones, IoT devices, surveillance devices, etc. As shown in Figure 2, a standard indoor setting has many links among them, one is main Line-Of-Sight (LOS), and many None Line-Of-Sight (NLOS) reflected paths due to the surroundings such as roof, pavement, and walls. The LOS path is affected by free-space path loss. According to the free space model, the power obtained by a receiver antenna is isolated from a radiating transmitter antenna by a distance *d*. Due to this, home networks have a vast number of WiFi links, as shown in Figure 2a. RSSI is the received signal strength and the path loss coefficient ($PL_{0}$) to estimate distance, as shown in Equation (1). The average RSSI for 10 s for the Figure 2a, is shown in Figure 2b. The CSI for the 30 sub-carriers, as shown in Figure 2c, follows a similar pattern, making it extremely useful for identifying gestures based on WiFi characteristics. Here $C_{0}=d_{0}^{\alpha }10^{\frac{-PL_{0}}{10}}$ is the average multiplicative gain at the reference distance $d_{0}$ from the transmitter and α is the path loss exponent. The path loss model can be expressed, according to Equation (2). The flat fading is represented by $F_{G}=10^{\frac{-X_{g}}{10}}$, $X_{g}$ is the attenuation caused by the flat fading. The distance is calculated from the received power. The RSSI shows the signal strength behavior in the coverage area and it shows the location estimation. However, the temperature can affect the transmission significantly. For the location feature, the accuracy of RSSI decreased with erroneous RSSI measurement.

(1) $$P_{R}=P_{T}\times \frac{C_{0}F_{G}}{d^{\gamma }}$$

(2) $$PL=P_{T}-P_{R}=PL_{0}+10\gamma log_{10}\frac{d}{d_{0}}+X_{g}$$

### 3.2. Channel State Information (CSI)

**Figure 3:**
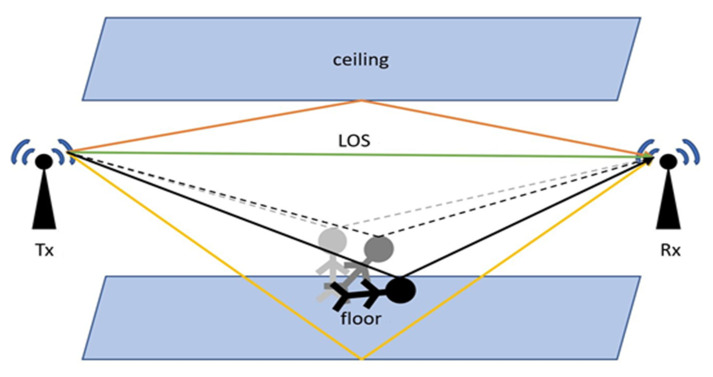
Signal propagation model in indoor environment.

Specifically, current WiFi standards (e.g., IEEE 802.11n/ac) use OFDM in their physical layer. In an indoor environment, WiFi signals travel through multiple paths, some of which pass through the reflection of the ceiling, the reflection of the floor, the reflection of the walls, etc. If there is a human inside the indoor environment, some extra paths, are caused by the reflection of the human body. Due to this, the received signal will change only when there is any human activity inside the indoor environment, as shown in Figure 3. This research will focus on using the signal changes caused by human action to detect the fall.

In wireless communication, CSI is a channel message in a communication link. Each link has a connection, the signal from the transmitting end to the receiving end through the channel such as scattering, fading, and attenuation of energy with distance. The CSI for the Figure 2a, is shown in Figure 2c. In the frequency domain, a narrowband flat-fading channel has multiple transmit and receive antennas that can be represented, as Equation (3). Where y and x are the receive and transmit vectors, respectively, and n is the noise vector, and H is the channel matrix. Here i is the subcarrier index, $x_{i}\in R^{N_{T}}$ and $y_{i}\in R^{N_{R}}$ are the transmitted signal and the received signal, respectively, $N_{T}$ is the number of transmitter antennas, $N_{R}$ is the number of receiver antennas, $n_{i}$ is the noise vector, and $H_{i}\in C^{N_{R}\times N_{T}}$ denotes the CSI matrix of the subcarrier *i*. We will have $N_{t}\times N_{r}$ pair transmit-receive antennas and $N_{t}\times N_{r}\times 30$ subcarriers for each CSI values.

(3) $$y_{i}=H_{i}\times x_{i}+n_{i}$$

(4) $$H_{i}=\left[\begin{array}{ccccc} \; & h_{i}^{11} & h_{i}^{12} & \ldots & h_{i}^{1N_{T}}\\ \; & h_{i}^{21} & h_{i}^{22} & \ldots & h_{i}^{2N_{T}}\\ \; & \vdots & \vdots & \vdots & \vdots \\ \; & h_{i}^{N_{R}1} & h_{i}^{N_{R}2} & \ldots & h_{i}^{N_{R}N_{T}} \end{array}\right]$$

(5) $$h\left(f\right)=\sum_{l=1}^{N}\alpha_{l}\exp^{-j2\pi f\tau_{l}}$$

where $h_{i}$ is the CSI of the *i*th subcarrier for the link between the receiver antenna and the transmitter antenna. Each CSI has amplitude and phase, influenced by the multi-paths, experience number of amplitude, and phase shift. Hence the CSI entry is represented as the channel frequency response (CFR) as shown in Equation (5). The CSI amplitude |h| and the phase $e^{sin\theta }$ are affected by the displacements and motion of the transmitter, receiver, and the nearby living and non-living things. In other words, we can say that CSI collects the wireless characteristics of the nearby environment. These wireless characteristics or the CFR are recorded for the number of subcarriers (i.e., 30) such as $h=[h_{1},h_{2},h_{3},...h_{30}]$. The CFR for the multipath scenario can be expressed by Equation (4). Here *N* is the total number of multipath, $\alpha_{l}$ and $\tau_{l}$ represent the attenuation and the propagation delay of the signal through path *l*, respectively. For each CSI entry, the CFR responses from all sub-carriers and transmission pairs are put together in one CSI matrix. Each frequency response is complex, expressed with amplitude and phase.

The CSI information can be received by using a computer with an IWL5300 NIC and using the Linux 802.11n CSI Tool [12]. Each pair of transmitting and receiving antennas will receive a set of CSIs containing 30 subcarriers. The denoising operation removes the noise from the received CSI information.

### 3.3. Denoising

As CSI is very sensitive to the environment, it is difficult to find some features extracted from the original CSI for action classification without removing the noise in the CSI. CSI contains amplitude and phase information; each of them has different denoising methods. There are several ways to denoise CSI amplitude, such as:

The weighted moving average (WMA) function computes the average of a set of input values over a specified number of periods represented by *n*. This function always gives greater weight to the recent data, accomplished by multiplying by a weighting factor. WMA can track more accurately than a corresponding Simple Moving Average due to its measurement. It helps in smoothening data series, which also reduces noise. The equation is Equation (6), here $Y_{t}$ is the received signal for t time stamps.

(6) $$WMA_{t}=\frac{n\times Y_{1}+\left(n-1\right)*Y_{2}+...Y_{t}}{\frac{n\times (n+1)}{2}}$$

Butterworth filter is more complex than WMA, designed to have a possible flat frequency response in the passband. The passband range is $\left[0,f_{c}\right]$, where $f_{c}$ is the allowed maximum low frequency above all to this, is blocked. This filter blocks the unwanted frequencies in the stop band. In Equation (7), where *m* is the order of the filter, the greater the order, steeper the response at the cutoff [39]. However, this filter shows poor phase characteristics.

(7) $$BWF\left(f,f_{c},m\right)=\frac{1}{1+{\left(\frac{f}{f_{C}}\right)}^{2m}}$$

Principal Component Analysis (PCA) is used to denoise and reduce dimensions. It is an orthogonal linear transformation used to find a projection of all data into k dimensions, whereas these k dimensions are those of the highest variance. The idea of PCA mainly wants to analyze the source of variation with fewer data dimensions. In [17], the first principal component contains noise, so it needs a denoising operation. The next five ones remain for feature extraction.

Discrete Wavelet Transform (DWT) is any wavelet transform which is discretely sampled, and has temporal resolution which can capture both frequency and location information. DWT uses wavelet filtering of different scales at each level and down sampling. The DWT can be expressed as Equation (8), here the signal x(t) is sampled to obtain discrete sequence $\psi (\frac{t-\tau }{a})$ is the mother wavelet. The values of parameters τ (shift) and *a* (scale) are $2^{j}$ and $n2^{j}$ respectively. the decomposition level is represented by *j* [40].

(8) $$DWT_{x(k)}\left(j,n\right)=\sum_{k=-\infty }^{\infty }x\left(k\right)\frac{1}{\sqrt{2^{j}}}\psi \left(\frac{k}{2^{j}}-n\right)\;\;\;j,n\in Z$$

Discrete Wavelet Denoising (DND) [18,41] has the high-frequency resolution but low time resolution at low frequencies and the high time resolution but low-frequency resolution at high frequencies. This feature makes DWT be able to remain the sudden signal changes and good signal resolution while denoising. The purpose of DWT denoising is to analyze data to discover hidden information.

As to the CSI phase, the most common way to denoise is Phase Sanitization [36,42]. Due to the defect on the hardware, there are errors with carrier frequency offset and sampling frequency offset. By using Phase Sanitization, the effect of phase errors can be eliminated.

### 3.4. GoogLeNet

GoogLeNet is a 22-layer deep convolutional neural network designed by Google researchers as a version of the Inception Network, a Deep Convolutional Neural Network, to maximize computational efficiency. In each block, it allows the network to choose from a variety of convolutional filter sizes. The concept behind this architecture, execute on individual devices with limited computing resources. Image recognition and target identification were among the computer vision tasks it solved [43].

### 3.5. NVIDIA DIGITS

NVIDIA’s DIGITS interface tools for neural networks. DIGITS allows developers to build end-to-end neural networks by importing datasets, splitting them for training and testing, and training the model. A user can improve accuracy by adjusting parameters such as bias, neural activation mechanisms, pooling windows, and layers. It has the advantage of allowing you to train multiple networks on the same data set or the same network on multiple datasets. With the GPU selection option, you can choose which GPUs to use for training each data set, making it easier to multitask with your hardware [44]. Due to the following reasons, we have decided to use this interface for our research.

### 3.6. Feature Extraction and Action Classification

In the non-deep learning method, most of them extract the features that can represent the waveform from the amplitude or phase information in the CSI. For example, WiFall integrates the amplitudes of the CSI into a stream and extracts seven features from the stream [13]. In AntiFall [14], authors select four subcarriers among 30 subcarriers and extract seven features from both amplitude and phase of CSIs. In the deep learning method, we can use the raw amplitude and phase of CSI as the input to the classification problem. To classify the action, Support Vector Machine (SVM) is a very popular classifier [13,14,15,18]. It can map the low-dimensional space to the high-dimensional space and nonlinear classification.

Deep Neural Network (DNN) [19] considers CSI as the two-dimensional input of amplitude and time CNN [20]. The CNN can learn its features automatically, although traditional approaches cannot. According to the study [17], the number of multi-paths obtained varies as the environment changes. However, the rate of change in multi-path length is fixed, and only the behavior of the human body causes this change, as seen in Figure 3. As a result, the frequency components in the CFR will remain constant as long as the human body performs the same action. This motivates us to use time-frequency analysis as input to the CNN for action classification. Some studies using DNN [19] or CNN [20] to classify CSI, but none of them can specifically extend their trained model to a new environment directly.

## 4. The Four-Stage Proposed Mechanism

**Figure 4:**
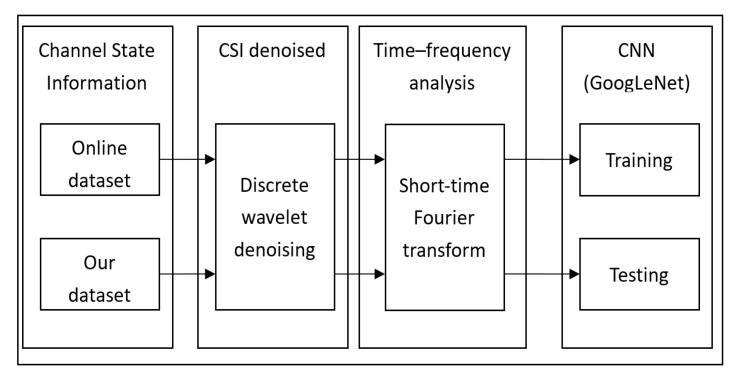
Four-stage system architecture.

In this section, we discuss the four-stage system architecture for our proposal as shown in Figure 4. In the first stage, we collect the WiFi characteristics for the different actions such as walk, sit down, fall. The CSI information for each action, shows the WiFi characteristics. The CSI dataset was collected from two sources: online and our. In stage two, the objective is to denoise the CSI signal using DND. The DND can show abrupt signal changes with a high signal resolution, which is very critical for our study. In stage three, we perform a short-time Fourier transform (STFT), to evaluate the sinusoidal frequency and phase content of local portions of a signal as it varies over time. The STFT splits the longer time signal into shorter segments and then performs the Fourier transform separately on each segment. Through doing so, the discrete data is transformed into a continuous data form at different frequencies, resulting in CSI time-frequency diagrams. Finally, in the fourth stage, we train our method with the help of GoogLeNet, using these time-frequency diagrams.

### 4.1. Stage Two: Denoising

To maintain the complete change of the signal, we use DND, which is the same as [18]. For signal denoising, an orthogonal wavelet, such as a Symlet or Daubechies is a suitable alternative. The Symlet wavelet has the least asymmetric wavelet and phase, while the Daubechies wavelet has a nonlinear phase, and the concentrated energy is at the beginning. The main distinction is that Symlet-4 (sym4) is used as our wavelet. Because of the higher symmetry, Symlet has an advantage over Daubechies for reducing distortion when processing and reconstructing signals. This method is very useful for increasing SNR and minimizing mean square error.

### 4.2. Stage Three: Short-Time Fourier Transform

A human action will change the lengths of multi-paths, but the frequency variable may not change as long as the same action [17]. If we do the same action in different locations, we get identical CSI time-frequency diagrams by converting the signal to the frequency domain. A Fourier-related transform is the STFT, works by dividing a longer time signal into shorter segments of equal length and then computing the Fourier transform independently for each shorter segments, allowing us to detect the frequency variable over time.

### 4.3. Stage Four: GoogLeNet

We can see from the time-frequency diagrams of CSIs that drastic action causes the high energy in high frequency in the spectrum. A fall produces a large amount of energy, at a high frequency in the spectrum for a short period. CNN is an appropriate tool for classifying time-frequency graphs, and the spectrum of different actions has different features. CNN does well in image recognition since it can quickly learn the features in the training phase. After turning CSIs into time-frequency diagrams, we can treat it as an image and input it into CNN. It is difficult to train a good CNN model with the fewer data provided by [16] and our collected data because CNN needs many images to train. Because of this difficulty, this research will use the pre-trained model to train the data. To alleviate the problem, we use the most widely known pre-trained model GoogLeNet which is on NVIDIA DIGITS as our method.

## 5. Evaluation

In this section, we introduce our experimental setup and the datasets. Then compare the results with other methods. We first compare the difference between CNN use and SVM and DNN use, where CSI amplitude is used directly as the input at the same place. It demonstrates that our approach can retain excellent accuracy when measured in various places, thus showing that converting the CSI to the time-frequency diagram can minimize CSI’s environmental sensitivity significantly.

### 5.1. Experiment Setup

**Figure 5:**
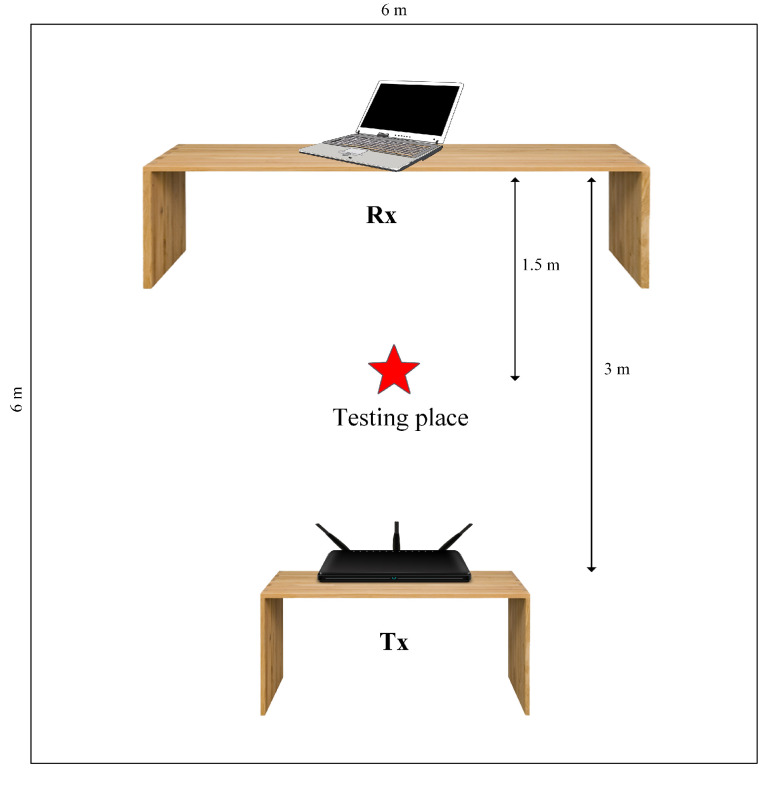
The experimental setting in the conference room.

As seen in Figure 5, we placed the laptop with Intel 5300 NIC 3 m away from the commercial AP in the 6 m × 6 m conference room, with the AP as Transmission (Tx) and the laptop as Receiver (Rx) in LOS. On different days, we gathered CSI of four actions, namely, a fall, a sit-down, a walk, and a bed, from five different individuals. We set the sampling rate of 1 kHz, and a human acts in the LOS state from both Tx and Rx, which was identical to [16]. We segmented the data after collecting it to remove the human action component. Each of the five persons should have performed more than 10 falls, sat more than 20 times, walked more than 30 times, and gone to bed more than 30 times. Since the data we gathered could only be used to validate the algorithm, there was no need to repeat the procedure as long as it would demonstrate that our method can achieve exceptional accuracy when tested in a different place.

During experiments, we used all the necessary safety equipment, such as crash mads, crash arm pads, and crash elbow pads. To avoid injuries to the lab members, we removed unnecessary furniture, such as tables and chairs, from the room. Our laboratory directors, Professor Shih-Lin Wu and Dr. Lokesh Sharma supervised all experiments.

### 5.2. Datasets

Although the dataset used to train the model [16], contained six actions: bed, fall, walk, run, sit down, and stand up, we only used four of them: bed, fall, walk, and sit down. This is because run and stand-up actions had the lesser number of entries in the open dataset. We then removed the CSIs and used DND to denoise, then used STFT to obtain the time-frequency diagram, which we then fed into GoogLeNet. Furthermore, owing to a shortage of data sets, we employed a process similar to Image Augmentation. If the diagram was cut at random, the action was likely to be omitted, and the data were devoid of action. As a result, we collected data that contained 0.1 s before and after the operation. This maximized the number of data points, and the data points also contained the whole action. We collected data from five different volunteers by having them perform the four acts on various days, and then we utilized the same approach without Image Augmentation to generate the time-frequency diagram and fed it into GoogLeNet, but simply to validate the method.

#### 5.2.1. Denoising

**Figure 6:**
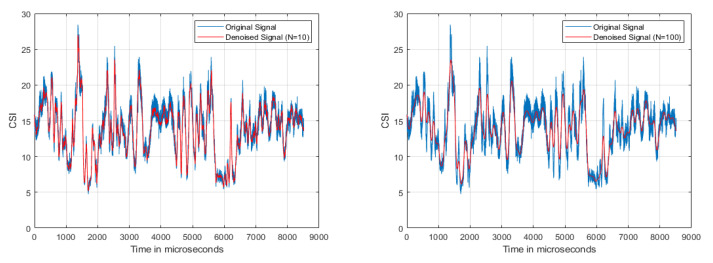
Weighted moving average filter.

**Figure 7:**
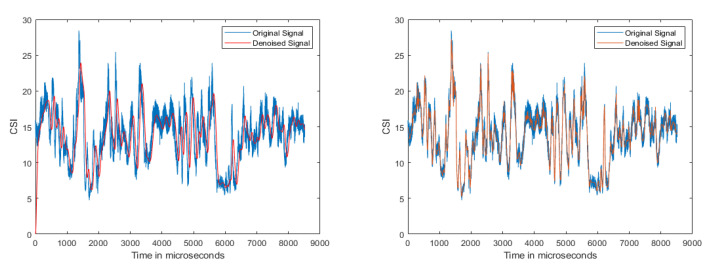
Other filters.

**Figure 8:**
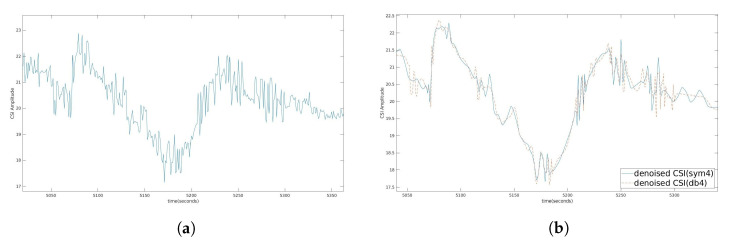
(**a**) Original CSI amplitude; (**b**) Denoised CSI amplitude for sym4 and db4.

Figure 6a,b demonstrate the two weighted moving average filters used to denoise for periods of n = 10 and 100, respectively, similar to [13]. The drawback was that some of the data used to compute the moving average might have been old. The Butterworth filter [22] denoised the signal as shown in Figure 7a; however, the filter also reduced the crests and troughs of the signal. To maintain the complete change of the signal, we implemented DND as shown in Figure 7b. Symlet is an improvement of Daubechies because of the higher symmetry, so it can be easier to reduce distortion when analyzing and reconstructing signals, as in Figure 8b. The original CSI amplitude was as shown in Figure 8a.

#### 5.2.2. Short-Time Fourier Transform

**Figure 9:**
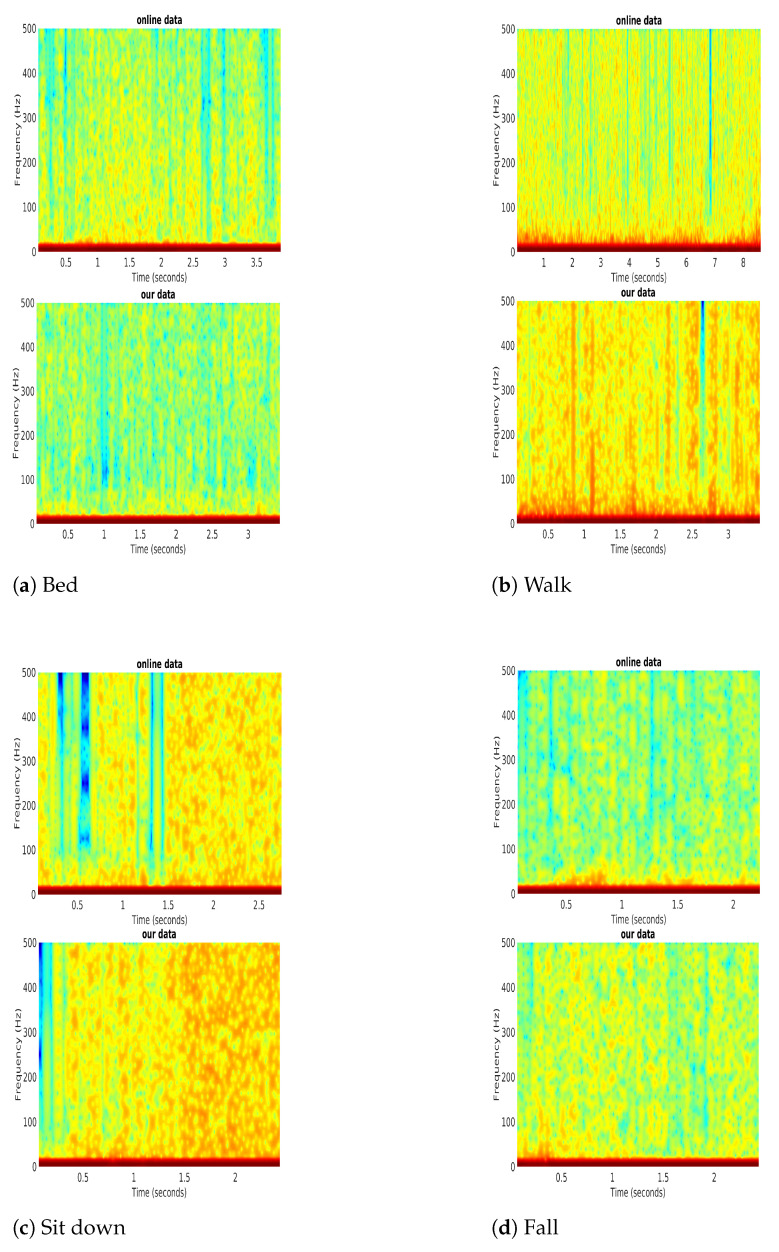
The Short-time Fourier Transform for different actions.

From the previous stage, we had the Symlet-4 denoising results for the four actions. The STFT provided FFT results for each action. To validate the STFT results, we compared our data with the online data, as shown in Figure 9. An action performed by humans will cause the changes in lengths of multi-paths, but the frequency component will not change as long as the same actions are performed [17]. Even if the same actions are performed in different places, we can still get similar CSI time-frequency diagrams. The similarity of the spectrum can be seen for different activities such as bed, walk, sit down, and fall as shown in Figure 9a–d respectively.

### 5.3. System Performance and Comparison

We compared the accuracy of CNN, DNN, and SVM while testing in the same and different places. Choosing SVM for comparison was because the study [13], i.e., the first research using CSI to detect fall employed the SVM to classify actions. We used the following eight features as input to SVM: (1) variance, (2) Normalized STD, (3) Median Absolute Deviation (MAD), (4) Interquartile Range (IR), (5) Signal Entropy, (6) Offset of Signal Strength, (7) mean of Velocity of Signal Change, and (8) max of Velocity of Signal Change. Furthermore, the difference between CNN and DNN was the convolution and pooling part before the fully connected layer, which was the process of CNN learning the features automatically. We want to emphasize the same action had a similar time-frequency diagram, even humans performing at different places, and we could use this to reduce the influences caused by the different places.

### 5.4. Test at the Same Place

**Figure 10:**
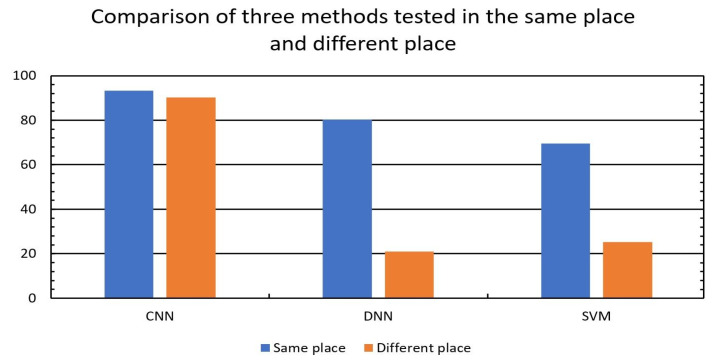
Accuracy comparison of three model between testing in the same environment and different environment.

For each method, we used the five-Fold Cross-Validation method to verify the accuracy. First, used the online data [16] to train and test our method, and the accuracy of bed, fall, walk and sit down are 95.6%, 94.4%, 98%, 84.7%, respectively, and average accuracy was 93.2%, as shown in Table 1. On the other hand, the average accuracies of SVM and DNN were respectively 80.3% and 69.5%, as shown in Figure 10. There are two reasons why DNN had lower accuracy than SVM. First, the input of the DNN was denoised CSI, and DNN could not learn the feature from the time-frequency diagram. Second, the number of online data [16] were not enough to train the DNN. If we had enough data, the performance of DNN would be better than SVM [19].

### 5.5. Test at the Different Places

The main difference between our method and others is that we turned CSI into the time-frequency diagram and used it as an input to the CNN model. As we have mentioned in Section 3, the time-frequency diagram of CSI will be similar as long as human performs the same action even at different places. As a result, when we tested our method in a different location, we could still get good results. Because CNN learned features from diagrams, the impact of performing in a different location was greatly reduced. Table 1 shows that our average accuracy still had 90.3%, and the accuracy of bed, fall, walk and sit down were respectively 91.6%, 90.3%, 97.5%, and 81.8%. However, the average accuracy of SVM and DNN decreased to 25.3% and 21.1%, respectively as shown in Figure 10. The features of the frequency components in the CFR remained the same as long as humans performed the same action, but the CSI amplitude could not be the same. Due to this, the accuracy of SVM and DNN dramatically decreased while testing at different places.

### 5.6. Discussion

**Figure 11:**
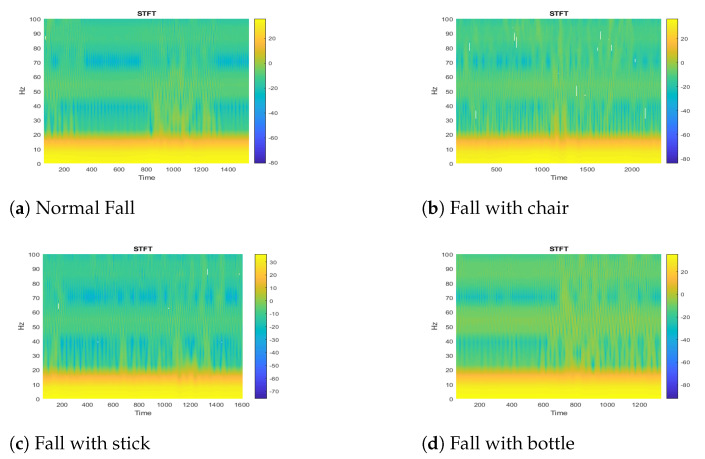
Elder person fall with different objects.

In this section, we have analyzed the CSI time-frequency diagrams of falls with different objects such as a chair, stick, and bottle in a static environment. While doing these experiments, our aim is to check the difference in WiFi characteristics, in an extreme attempt to hold on when an elderly person is falling. In Figure 11, we evaluate the CSI and STFT diagram with possible holding objects. Figure 11a–d shows a person fall in normal, holding a chair, with a stick, and with a bottle conditions. The CSI time-frequency diagrams show that there is no effect in WiFi characteristics even in the extreme attempt of holding objects.

## 6. Conclusions

The low accuracy of WiFi-based fall detections is the subject of this investigation. Previously, the system is trained at the original signal collection/training place, but the application is executed in a different place with different WiFi characteristics. This results in poor accuracy. Our proposed solution demonstrated that after converting CSIs into a time-frequency diagram, the same actions have similar characteristics even when conducted at different locations, and we can use these diagrams as input to the CNN to reduce the effect of different locations. We compare our method’s accuracy to that of SVM and DNN in the same and different places. When tested in the same context, our method’s accuracy is only marginally higher than that of SVM and DNN; however, when tested in a different context, our method’s accuracy stays about 90%, which is significantly higher than other techniques. We believe that acquiring additional data from more diverse sources will improve accuracy when assessing our method in a location to turn from the training data, allowing device-free fall detection systems to be more efficient.

## Figures and Tables

**Table 1:** Comparison of the accuracy when testing in the same and different environment.

Accuracy (%)	Bed	Fall	Walk	Sit Down	Average
Same Place	95.6	94.4	98	84.7	93.2
Different place	91.6	90.3	97.5	81.8	90.3

## Data Availability

Not applicable.
